# Constitutional Effects of Anæsthetic Agents

**Published:** 1857-04

**Authors:** 


### ARTICLE XI.

### *Constitutional Effects of Anæsthetic Agents.*

The *New York Journal of Medicine* (Sept. 1856) contains an interesting paper on this subject, by Dr. J. Henry Clark, of Newark, N. J., in which he relates the three following cases occurring in his practice, where he considers that the anæsthetic, without producing any untoward effects at the time of administration, gave rise subsequently to a train of symptoms of a very distressing character:

"*Case* 1.—Mr. J. M., a gentleman about 30 years of age, of good constitution, who ordinarily enjoyed good health, took *ether*, to facilitate the extraction of a tooth. He is of a nervous temperament, light complexion, blue eyes, and light hair. The administration of the agent produced no unusual effect, except that a 'choking feeling' was experienced just before the occurrence of complete insensibility. After recovery from the immediate effect, for several weeks extreme nausea and constant pain in the head were experienced.

"After the lapse of three or four months, Mr. M. applied to me for advice, complaining of the following train of symptoms, the commencement of which he dated from the day that he inhaled the ether. He is an intelligent man, and has no doubt that they are wholly attributable to the inhalation, and that they commenced at that time. These symptoms, he says, he never before experienced. Is very 'bilious' (to use his own language;) tongue much coated; constant pain in the head, especially over the eyes; a 'feeling as if there was a *want of mental exercise;*' habitual costiveness; pain in the right side and back; indigestion; want of muscular strength; nervous system greatly deranged; neuralgic pains; and great despondency of mind.

"Change of habits, mineral tonics, and alteratives restored him to perfect health after a few months. All these symptoms at length disappeared.

"*Case* 2.—Mr. C. M. inhaled *chloroform*, for the purpose of having a tooth extracted, in November, 1854. In the afternoon of January 5, 1855, I was called hastily; found him in a state of dreadful nervous excitement. He had just recovered from a period of insensibility; his extremities were cool; pupils of the eyes natural; some heat about the head and neck; convulsive twitchings in the face; and trembling, with nervous agitation and mental excitation.

"When the urgent symptoms were relieved, I learned that he recovered from the immediate effects of the inhalation without any unusual symptom; that it was followed by a very unpleasant feeling in the top of the head; such as he had never experienced before; that this symptom and neuralgic pains had been constantly experienced from the day that he took the chloroform.

"In the morning of January 6, while lying reading on the sofa, he was seized with a singular sensation of bewilderment and other feelings that he finds no language to describe. These sensations greatly alarmed him. Recovering himelf, he went to his father's warehouse; while there engaged, an hour or two afterwards, he again experienced those singular sensations, but found himself unable to *articulate distinctly.* His father perceiving that he was ill, observing convulsive twitching in his face, with pallor and anxiety, and perceiving in his conduct a desire to go home, took his arm and walked with him to his residence, about half a mile distant. I was soon after summoned, and found him in the condition heretofore described.

"I prescribed stimulants, anti-spasmodics, and alteratives. He did not leave his room for a week, during which he suffered from extreme restlessness, anxiety and vertigo. His pulse continued, during the week, depressed. I treated him with tonics and anti-spasmodics, and mild mercurial alteratives, with evident advantage; but his neuralgic symptoms yielded slowly. His liver was constantly torpid; lassitude, indecision, and depression of spirits were indicated in his conduct.

"I advised a voyage to Europe. He sailed in May, and remained till September. He returned very much benefited, but had the same furred tongue that was evident in the case previously referred to, with more or less habitual costiveness. These symptoms have yielded to the use of mild alteratives, and he seems now, after the lapse of fifteen months since its inhalation, to have recovered again his wonted strength and vigor.

"In the case last alluded to, it required about eighteen months to rally from the influence of the ethereal inhalation. In the case to which I shall next refer, over two years was required to outlive the same train of symptoms, mostly neuralgic, which commenced from the day of the inhalation.

"*Case* 3.—Mrs. W., about 25 years of age, dark complexion, dark eyes, dark hair, of sanguine temperament, none of the nervous. She had usually enjoyed good health, although not robust. She received *chloroform* at the hands of a dentist also. I was called a year afterwards to prescribe for a set of symptoms very much like those described in the cases before narrated; neuralgia and 'strange feelings about the head' were the prominent symptoms described, with a general feeling of 'malaise.' I found as in the other cases, a determined conviction that all these symptoms were new, and that they commenced with the inhalation. I treated her as I did the others, depending mainly upon *iron ;* the appearance of the countenance indicated the want of a larger supply of iron in the blood. She gradually regained her usual health. It required a full year, however, to accomplish the cure. The writer believes that the experience of others, if it could be accumulated, would prove that—

"1. Chloroform is most dangerous when employed in cases of trifling importance, both in relation to its immediate effects and final results. This is proved by the greater frequency of cases in which unpleasant results are observed, which follow the extraction of teeth, or its use in trifling operations, where there is not pain enough to resist its excessive effects.

"2. That persons whose nervous systems are particularly susceptible are most liable to suffer from the inhalation of these agents.

"3. That the use of chloroform in the lying-in chamber is not devoid of danger.

"A very limited inquiry into the experience of others has been made, with the following results. An eminent practitioner, in answer to my inquiry, says, 'I have given it in four cases of accouchement. One of my patients had puerperal mania, and another congestion of the brain, both within six days afterwards. It is possible that chloroform was not the cause in either case ; but I have never ventured to use it since.' A lady of eminence in New Jersey took chloroform in labor, became comatose, immediately recovered, with *permanently impaired* intellect.

"In the experience of a neighboring practitioner, a perfectly healthy girl, aged 18, sanguine temperament—catamenia regular—applied to a dentist to have a tooth extracted under the influence of chloroform. It was done very carefully by a prudent man. A very few inspirations sufficed to produce insensibility. The tooth was extracted, but sensibility did not return for several days; a serious congestion of the brain followed, continuing for several weeks, and though several years have elapsed, she continues in a partially demented condition, subject to periods of excitement somewhat like those which she exhibited during the original attack.

"A physician of New York city relates a case of a young woman, aged 28, in good health, who after the extraction of a tooth, was immediately seized with the most distressing symptoms. Every muscle in the body commenced jerking and twitching. These phenomena continued for four days, despite the means adopted for her relief. They ceased at length under the use of strychnia. I am not informed with regard to the influence upon the mind.

"The same gentleman related the case of a young girl, in a good family, (without date, age, name, or any facts, lest the matter might become public and grieve the friends,) who became crazed immediately after taking chloroform to facilitate the extraction of a tooth. *Years have passed, and she is still a maniac.*

"Another case has come to my knowledge of a child fourteen years old, who took chloroform when about to undergo a trifling operation. She became deranged. *Six years have passed, and she has in nowise recovered.*

"Morris County, N. J., furnishes a case that I am not at liberty to report, in which permanent idiocy resulted from the inhalation of chloroform.

"That these agents should be used with great caution is further proved from their influence upon certain susceptible constitutions, when not taken for the purpose of producing insensibility, but when merely exposed to an atmosphere charged with the fumes of ether or chloroform. On this point I have several illustrations, but one will suffice.

"Mr. ——, a highly respectable dentist of——, of nervous habit, light complexion, active motion, suffered from all the symptoms which were observable in the cases which I reported in my own experience in the early part of this article, simply from breathing the fumes of these anæsthetic agents as they permeated the apartments in which he operated and resided. He was accustomed to use these agents in his practice almost hourly. His house was perpetually filled with the odor. He became at length feeble, pulseless, anemic—'felt as if he had been drinking champagne'—suffered from neuralgia and very great nervous debility. Abandonment of business, a residence in the country for three years, and constant out door exercise, and the use of electricity, accomplished a cure at the *end of that period.* He has no doubt but his illness resulted from this cause, and that his recovery is due to its removal, and the remedies employed.

"My honored and beloved preceptor, the late James C. Bliss, M. D., related a case to me last spring, in which amaurosis, followed by idiocy, resulted from the use of chloroform or ether in parturition. They succeeded the inhalation so promptly, that it is evidently proper thus to trace cause and effect. I understood that all who saw her, both in and out of the profession, attributed the amaurosis and the subsequent derangement to the inhalation of one of these agents—I did not understand which.

"Several other cases have come to my notice, in which the inhalation of anæsthetics seemed to produce similar constitutional effects. They were not my own patients, and I could not get access to well-authenticated facts in relation to them.

"These facts have made upon my own mind the impression that these agents should be regarded as remedies of great potency, and not always certain to produce none but salutary effects; that they should not, if possible, be administered to persons subject to local determination, or where their antecedents offer reason to apprehend derangement; that their use should be avoided in persons of extremely nervous temperament, or for any cause of nervous susceptibility.

"We have the authority of eminent men, in Europe and in this country, whose experience in the use of these agents has been very extensive, in favor of their usefulness and safety. Still, these gentlemen have committed themselves in favor of what may be regarded as their hobby, without being chargeable with any want of respect, and the experience of others does not justify fully their opinions with regard to their entire safety.

"If cases of dying do *sometimes* occur in the lying in chamber and on the operating table—if debility, hepatic congestion, neuralgia, apoplexy and mania do *sometimes* follow their use, it would appear proper that he who would deserve a reputation for wisdom and discretion should be quite certain that the severity of the case demands a *somewhat hazardous* remedy. Chloroform furnishes most valuable assistance in the operations of surgery and the lying-in chamber. With no disposition to undervalue it, and far less to treat lightly the valuable statistical evidence furnished by Professor Channing, of Boston, and Professor Simpson, of Edinburgh, of its comparative safety when carefully employed, I canuot believe that its emyloyment to relieve the pangs of *ordinary confinement*, or to allay the pain of a trifling operation, is wise or expedient. If in my comparatively limited experience three cases should come under my observation within about two years, others, situated more advantageously, could note many such cases, if looked for. To record the result of my observations, and to prompt abler observers to look in the same direction, is the whole of my present purpose.

"While writing the concluding pages of this article, I have been called to administer chloric ether in a case of amputation of the adventitious toe of each foot from a young gentleman of vigorous health. Although the operation was not very protracted, and the ether was administered by a discreet medical assistant, derangement followed; nor were my anxieties entirely relieved with regard to these effects before the lapse of six er eight hours.

"If the facts which I have collated are sustained by those of larger experience—if there are constitutional effects as well as immediate to be guarded against, more regard should be given to the habits of the patient, and these agents should be reserved for grave and trying emergencies."

